# Assessment of health-related quality of life of Bangladeshi patients with type 2 diabetes using the EQ-5D: a cross-sectional study

**DOI:** 10.1186/s13104-015-1453-9

**Published:** 2015-09-29

**Authors:** Farzana Saleh, Ferdous Ara, Shirin Jahan Mumu, Md Abdul Hafez

**Affiliations:** Department of Community Nutrition, Bangladesh University of Health Sciences (BUHS), 125/1 Darussalam Mirpur 1, Dhaka, 1216 Bangladesh; BRAC Institute of Global Health, BRAC University, icddr,b campus, 68 Shaheed Tajuddin Ahmed Sharani, Mohakhali, Dhaka, 1212 Bangladesh; Department of Epidemiology, BUHS, Dhaka, Bangladesh; Department of Biostatistics, BUHS, Dhaka, Bangladesh

**Keywords:** Cross-sectional study, EQ-5D, Health-related quality of life, Quality of life, Type 2 diabetes, Bangladesh

## Abstract

**Background:**

The management of diabetes requires a fundamental change in the lifestyle of patients, and one of the important outcome criteria is the quality of life. We assessed the health-related quality of life (HR-QoL) and examined the factors associated with it in type 2 diabetes.

**Methods:**

An analytical cross-sectional study was conducted among 500 type 2 diabetes patients (age >25 years and duration of diabetes >1 year). They were selected conveniently from the Out-Patient department of the Bangladesh Institute of Health Sciences Hospital. The HR-QoL was assessed using an adapted and validated Bangla version of the EQ-5D (© 1990 EuroQol Group. EQ-5D™) questionnaire. It has five domains: mobility, self-care, usual activities, pain/discomfort, and anxiety/depression and two levels (problem and no problem) on each dimension. The responses to the EQ-5D were further translated into a single summary EQ-5D index using the UK TTO value set.

**Results:**

Of the patients, 50.2 % were female, and 49.4 % were aged >55 years. Only 28.4 % had completed higher secondary education, and 50.8 % were from lower-middle-income families. Around 78.8 % either had overweight or were obese. About 50.4 % had problems in mobility, 28.2 % in self-care, 47.6 % in usual activities, 72.8 % in pain/discomfort, and 73.6 % in anxiety/depression. Results of binary logistic regression analysis showed that age, gender, lower-middle income, and HbA1_C_ were significantly (p < 0.05) associated with mobility. Self-care was significantly (p < 0.05) related to age, family history and duration of diabetes mellitus (DM). Gender, family history of DM, and lower-middle income had a significant (p < 0.05) association with usual activities. Pain was significantly (p < 0.05) associated with age, lower-middle income, and upper-middle income. Rural area, higher education, and HbA1_C_ were significantly (p < 0.05) related to anxiety. Results of multiple linear regression analysis showed that age (p = 0.0001), female gender (p = 0.0001), and prescribed treatment (p = 0.048) were associated with the EQ-5D index.

**Conclusions:**

The large majority (73 %) of the patients had problems in pain/discomfort and anxiety/depression; 50 % had problems in mobility and usual activities; and three in ten in self-care. Age, female gender, income, education, family history and duration of DM, and prescribed treatment are important factors that are associated with the HR-QoL in type 2 diabetes.

## Background

Diabetes is a chronic disease with a considerable impact on the health status and quality of life and is considered an urgent public-health issue because of its epidemic perspective. According to the World Health Organization (WHO), compared to the developed world, developing countries have a larger burden of diabetes [1]. Due to its low awareness among the public in developing countries, it is certain that they will face the impact of diabetes waves in coming years. Now a day, the number of type 2 diabetes people is increasing in every country, and in every 6 s, a person dies from diabetes [2].

Globally, the majority of the 382 million people with diabetes are aged 40–59 years; 80 % of them live in low- and middle-income countries; and the percentage of people with type 2 diabetes will increase to 55 % in 2035 [2].

Like all other developed and developing countries, the prevalence and the incidence of type 2 diabetes mellitus (DM) are also increasing in Bangladesh. In 2013, its prevalence in Bangladesh was estimated to be 7.11 % by the International Diabetes Federation (IDF) [2]. By 2030, the number of diabetes patients is expected to rise to 11.1 million; this explosion will place the country among the top seven countries of the world in 2030 [3].

The WHO in 1948 defined health from a new perspective, not only by the absence of disease and infirmity, but also by the presence of physical, mental and social well-being [4]. The quality of life is an important health outcome in its own right, representing the ultimate goal of all health interventions. Clinicians and public-health officials have used the health-related quality of life (HR-QoL) and well-being to measure the effects of chronic illness, treatments, and short- and long-term disabilities [5]. Persons with diabetes have lower quality of life than persons without chronic illnesses; however, the quality of their life is better compared to patients with other serious chronic diseases [6].

A complete cure of chronic diabetes cannot be achieved, and as such, it has a considerable impact on key aspects of patients’ well-being. Although clinical measures provide a good estimate of disease control, the ultimate objective of diabetes care is to improve the patient’s HR-QoL.

The EuroQol 5D (EQ-5D), a generic measure of HR-QoL, is widely used for determining the quality of life. Some studies have as well used it for estimating the HR-QoL of patients with type 2 diabetes in Western and Asian countries. The FIELD study reported that that the EQ-5D index is an independent predictor of mortality risk, future vascular events, and other complications in patients with type 2 diabetes [7]. The EQ-5D was used in Japan [8] and Korea [9] for measuring the HR-QoL in patients with type 2 diabetes.

Several studies have identified several factors that influence the HR-QoL in patients with diabetes [8–13]. The measurement of HR-QoL indicates a comprehensive evaluation of the patient’s health status which would provide additional information to laboratory data and subjective symptoms.

Awareness of diabetes among patients in developing countries like Bangladesh is still poor [14]. Despite its high prevalence and the importance of HR-QoL in the management of diabetes, little is known about the HR-QoL of patients with diabetes in Bangladesh. Against this background, the present study was conducted to assess the HR-QoL of Bangladeshi patients with type 2 diabetes.

## Methods

This analytical cross-sectional study was conducted among 500 patients with type 2 diabetes. They were selected conveniently from the Out-Patient department (OPD) of the Bangladesh Institute of Health Sciences (BIHS) hospital. The minimum required sample-size was calculated using the formula n = z^2^ pq/d^2^; where, z = 1.96, p = the expected rates of problem in health-related quality of life, i.e. 31 % [9]; q = (100 − p), and d = allowable error of known prevalence, i.e. 4 %. Patients who were aged >25 years, having diabetes for at least 1 year (from the date of interview) were included in the study. Patients who had other medical complications or who were unable to answer a short list of simple questions (sociodemographic information, such as name, address, disease complications, etc.) were excluded from the study.

### Instruments

An interviewer-administered questionnaire was designed to know and assess the level of the patient’s HR-QoL. Information on age, sex, educational qualification, occupation, monthly income, duration of diabetes, family history of diabetes, acquisition of information relating to diabetes, and prescribed treatment for patients was collected by interviewing the patients. A checklist was used for collecting HbA1c data from the patients’ guidebook. The checklist means an instrument used when observing some situation. The researcher/interviewers put tick marks against the particular point or wrote down what he/she observed.

### Anthropometric measurements

Anthropometric measurements included weight and height of the patients. Body-weight in light clothes was measured to the nearest 0.1 kg using a Sohenle mechanical weighing scale (Soehnle-Waagen GmbH & Co. KG, Wilhelm-Soehnle-Strabe 2, D-71540 Murrhardt/Germany). Height was measured to the nearest 0.5 cm using a portable, locally-manufactured stadiometer, with patients standing upright on a flat surface without shoes, and the back of the heels and the occiput remaining on the stadiometer. The questionnaire was pretested before its finalization.

### Measurement of HR-QoL

The HR-QoL was assessed using an adapted and validated Bangla (local language) version of the EQ-5D [15]. This generic instrument has five dimensions, such as mobility, self-care, usual activities, pain/discomfort, and anxiety/depression. Each dimension has three levels, such as no problem, some problems, and extreme problems. For binary logistic analysis, the level of each domain was divided into two levels, such as no problem and problems (by collapsing the other levels). However, according to the suggestion of the EuroQol group, sometimes it is more convenient to dichotomize the EQ-5D levels into ‘no problem’ (i.e. level 1) and ‘problems’ (i.e. level 2 and 3) [15]. In our study, we faced a very few number of reported level 3 problems.

The developers of the EQ-5D have generated value sets in several countries to calculate a preference-based index for the 243 health states defined by responses to the five questions of the EQ-5D, using a scale on which 0.0 represents being dead and 1.0 full health. Values of the index can be negative for states that are deemed to be ‘worse than death’: so for example, the minimum value in the UK-based value set is −0.59, which represents the worst possible health state (i.e. 33,333) [16]. As there is no value set based on time trade off developed for the South-East Asian population we have used the time trade off (TTO) method conducted in the United Kingdom [16] i.e., UK TTO most commonly used sets currently available for the EQ-5D [16].

### Socioeconomic classifications

The patients were grouped according to the 2006 per-capita gross national income (GNI) and the World Bank calculations [17] as follows: low income: US$ ≤905 or Tk ≤4906; lower-middle income: US$ 906–3595 or Tk 4907–19,488; upper-middle income: US$ 3596–11115 or Tk 19489–60252; and high income: US$ ≥11,116 or Tk ≥60,252.

### Analysis of data

Frequencies and mean ± SD were calculated for descriptive analysis. Binary logistic regression was used for examining the factors associated with the EQ-5D domains. The 12 independent variables used for each proposed model were: age, gender, habitat, education, occupation, income, family history of diabetes, duration of diabetes, acquisition of information, BMI, HbA1c, and prescribed treatment. Of these, seven independent variables were significantly associated with the EQ-5D domains, and only significant results are presented in the tables. Multiple linear regression was used for finding out the factors associated with the EQ-5D index. Statistical tests were considered significant at p value of ≤5 % (≤0.05).

In the study, acquisition of information defined as patients was obtained information on diabetes from physicians, nurses, health educators, and nutritionists and also from different types of tools, such as magazine, leaflet, guidebook, and TV shows. Prescribed treatment implied prescriptions made by the respective physicians.

## Ethical aspects

Informed written consent was obtained from all the respondents after fully explaining the nature, purpose, and procedures used for the study to them. Ethical approval was obtained from the ethics and research review committees of the Diabetic Association of Bangladesh.

## Results

Table 1 presents the sociodemographic and other relevant characteristics of the patients. Their mean age was 54.2 (±11.2) years. Of the patients, 50.2 % were female; 49.4 % were aged >55 years; 28.4 % had completed higher secondary education; 79.8 % were from the urban area; 29.8 % were employed; 46.6 % were homemakers; and 40.4 and 50.8 % were from upper-middle- and lower-middle-income families respectively. The large majority (70.4 %) of the patients had a positive family history of diabetes, and 47.8 % had diabetes for 5 years or less. Twenty-three percent did not get any information on diabetes from any source. The mean HbA_1_c (%) of the patients was 6.45 (±1.81). According to Asian BMI [18] cut-off, 52.8 % were at an increased risk and 26 % at a high risk. More than half (57 %) were prescribed for oral hypoglycemic agent (OHA) and 31 % for both insulin and OHA.

**Table 1:** Characteristics of patients (n = 500)

Parameter	
Age (years)	54.21 ± 11.22
25–39	39 (7.8)
40–54	214 (42.8)
≥55	247 (49.4)
Gender	
Female	251 (50.2)
Male	249 (49.8)
Education	
Illiterate	105 (21)
Primary to 8th grade	154 (30.8)
SSC to HSC	142 (28.4)
Graduate and above	99 (19.8)
Habitat	
Urban	399 (79.8)
Rural	95 (19)
Semi-urban	6 (1.2)
Occupation	
Service	78 (15.6)
Business	71 (14.2)
Homemaker	233 (46.6)
Others (labor/unemployed)	118 (23.6)
Monthly income (US$)	
High income (≥11,115)	35 (7)
Upper-middle income (3596–11,115)	202 (40.4)
Lower-middle income (906–3595)	254 (50.8)
Low income (≤905)	9 (1.8)
Family history of DM	
No	132 (26.4)
Yes	352 (70.4)
Do not know	16 (3.2)
Duration (years) of DM	
≤5	239 (47.8)
6–12	173 (34.6)
13–18	50 (10)
>18	37 (7.4)
Acquisition of information	
Yes	384 (77)
No	115 (23)
Prescribed treatment	
OHA	287 (57.4)
Insulin	44 (8.8)
OHA + insulin	155 (31)
Diet	14 (2.8)
HbA_1c_ (%)	6.45 ± 1.81
Body mass index (kg/m^2^)	26.1 ± 6.7
Underweight	5 (1)
Acceptable risk	99 (19.8)
Increased risk	264 (52.8)
High risk	130 (26)

Results are expressed as number (%) and mean ± SD; US$ 1 = Tk 77.47

*OHA* oral hypoglycemic agent

**Fig. 1:**
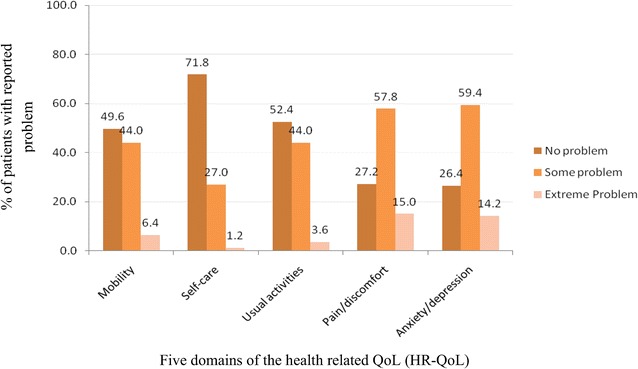
Profile of health-related quality of life among patients (n = 500)

The profile of the HR-QoL of the patients by domain is presented in Fig. 1. Figure 1 shows that about 44 % of the patients had some problems in mobility, 27 % in self-care, 44 % in usual activities, 57.8 % in pain/discomfort, and 59.4 % in anxiety/depression. Extreme problem in mobility was observed among 6.4 % of the patients, 1.2 % in self-care, 3.6 % in usual activities, 15 % in pain/discomfort, and 14.2 % in anxiety/depression.

Results of binary logistic regression analysis showed that the upper-middle-income group had two times likely to have problems in mobility compared to the other groups (β = 1.007; p = 0.022; odds ratio OR = 2.738; 95 % CI 1.160–6.463). Age showed a significant association (β = 0.039; p = 0.002; OR = 1.039; 95 % CI 1.014–1.065) with self-care. Having a positive family history of diabetes (β = −0.867; p = 0.001; OR = 0.420; 95 % CI 0.250–0.706) and the duration of diabetes (β = 0.042; p = 0.021; OR = 1.043; 95 % CI 1.006–1.082) showed a significant association with self-care. The female group had three times more likely to have problems in usual activities compared to the male (β = 1.322; p = 0.022; OR = 3.751; 95 % CI 1.207–11.658). The upper-middle-income group was two times likely to have problems with usual activities compared to the other groups (β = 0.974; p = 0.024; OR = 2.648; 95 % CI 1.136–6.173). The positive family history of diabetes also showed a significant association with usual activities (β = −0.767; p = 0.002; OR = 0.464; 95 % CI 0.288–0.747). Age showed a significant association (β = 0.050; p = 0.0001; OR = 1.051; 95 % CI 1.025–1.079) with pain/discomfort. The upper-middle-income group (β = 1.034; p = 0.015; OR = 2.811; 95 % CI 1.227–6.441) and the lower-middle-income group (β = 0.946; p = 0.023; OR = 2.576; 95 % CI 1.138–5.832) were two times likely to have problems with pain/discomfort compared to the other groups. The rural patients felt anxiety/depression significantly compared to the urban patients (β = −0.588; p = 0.042; OR = 0.555; 95 % CI 0.315–0.978). The higher education group showed a significant association with anxiety/depression (β = −0.960; p = 0.028; OR = 0.383; 95 % CI 0.162–0.902) (Table 2).

**Table 2:** Binary logistic regression for estimating odds ratio and 95 % confidence interval for assessing HR-QoL (with ‘no problem’ in five domains as the reference category) by selected factors

Independent variable	β	Sig.	Odds ratio	95 % CI for EXP (β)	
				Lower	Upper
a. Dependent variable: mobility					
Age (years)	0.057	0.0001	1.059	1.034	1.085
Gender					
Male	Reference				
Female	1.640	0.007	5.156	1.564	16.997
Monthly income (Tk)					
High-income group	Reference				
Upper-middle-income group	1.007	0.022	2.738	1.160	6.463
Lower-middle-income group	0.371	0.393	1.449	0.619	3.393
Low-income group	1.146	0.210	3.145	0.523	18.90
HbA1_c_ %	0.120	0.06	1.128	0.997	1.276
b. Dependent variable: self-care					
Age (years)	0.039	0.002	1.039	1.014	1.065
Family history of diabetes					
No	Reference				
Yes	−0.867	0.001	0.420	0.250	0.706
Don’t know	0.257	0.669	1.293	0.398	4.199
Duration (years) of diabetes	0.042	0.021	1.043	1.006	1.082
c. Dependent variable: usual activity					
Gender					
Male	Reference				
Female	1.322	0.022	3.751	1.207	11.658
Monthly income (Tk)					
High-income group	Reference				
Upper-middle-income group	0.974	0.024	2.648	1.136	6.173
Lower-middle-income group	0.384	0.371	1.469	0.633	3.41
Low-income group	0.337	0.696	1.401	0.258	7.609
Family history of diabetes					
No	Reference				
Yes	−0.767	0.002	0.464	0.288	0.747
Don’t know	0.618	0.335	1.855	0.528	6.520
d. Dependent variable: pain					
Age (years)	0.050	0.0001	1.051	1.025	1.079
Monthly income (Tk)					
High-income group	Reference				
Upper-middle-income group	1.034	0.015	2.811	1.227	6.441
Lower-middle-income group	0.946	0.023	2.576	1.138	5.832
Low-income group	0.188	0.836	1.207	0.205	7.115
e. Dependent variable: anxiety					
Habitat					
Urban	Reference				
Rural	−0.588	0.042	0.555	0.315	0.978
Semi-urban	20.449	0.999	7.60	0.0001	<0.001
Level of education					
Illiterate	Reference				
Primary to 8th grade	−0.577	0.085	0.562	0.291	1.082
SSC to HSC	−0.446	0.228	0.640	0.310	1.321
Graduate and postgraduate	−0.960	0.028	0.383	0.162	0.902
HbA1_c_ %	0.128	0.06	1.136	0.996	1.296

β for standardized regression coefficient. Level of practice was taken as a dependent variable whereas other variables were taken as independent variables

*HR-QoL* health-related quality of life

Twelve independent variables together (irrespective of significant or non-significant) showed about 31.4, 26.8, 22.4, 21.3, and 16 % (Nagelkerke’s R^2^) of variation in mobility, self-care, usual activities, pain/discomfort, and anxiety/depression respectively (Table 3).

**Table 3:** Status of association of independent variables with domains at a glance

Domain	Significantly associated variable	Not significantly associated variable	Nagelkerke’s R^2^
Mobility	Age, gender, monthly income	Habitat, family history of DM, duration of DM, education, occupation, acquisition of information, BMI, HbA1c, prescribed treatment	0.314 ≅31.4 %
Self-care	Age, family history of DM, duration of DM	Gender, habitat, education, occupation, acquisition of information, BMI, prescribed treatment, monthly income, HbA1c	0.268 ≅26.8 %
Usual activities	Gender, family history of DM, monthly income	Age, habitat, duration of DM, education, occupation, acquisition of information, BMI, prescribed treatment, HbA1c	0.224 ≅22.4 %
Pain/discomfort	Gender, monthly income	Gender, habitat, family history of DM, duration of DM, education, occupation, acquisition of information, BMI, prescribed treatment, HbA1c	0.213 ≅21.3 %
Anxiety/depression	Habitat, education	Age, gender, family history of DM, duration of DM, occupation, acquisition of information, BMI, HbA1c, prescribed treatment, monthly income	0.160 ≅16 %

Results of multiple linear regression analyses for the EQ-5D index are presented in Table 4, and the results showed that the overall multiple regression model achieved an adjusted R^2^ of 0.20; p = 0.0001. In this model, gender had an influence on the HR-QoL (p = 0.0001). Other sociodemographic factors, i.e. older age, were associated with the lower quality of life (p = 0.0001) and higher education with the better quality of life (p = 0.025). Prescribed treatment was also significantly (p = 0.048) associated with the quality of life.

**Table 4:** Multiple Linear regression analysis of EQ-5D index value as a dependent variable with other parameters of the patients (n = 500)

Predictor variable	B^a^ ± SE	Beta^b^	P value	95 % CI for B	
				Lower	Upper
Age (years)	−0.007 ± 0.002	−0.223	0.0001	−0.010	−0.004
Gender	−0.280 ± 0.033	−0.398	0.0001	−0.344	−0.215
Habitat	0.012 ± 0.035	0.015	0.732	−0.056	0.080
Education	0.007 ± 0.003	0.112	0.025	0.001	0.014
Occupation	−0.012 ± 0.016	−0.034	0.451	−0.045	0.020
Family history of DM	4.78 ± 0.0001	0.024	0.556	<0.0001	<0.0001
Duration (years) of DM	−0.003 ± 0.002	−0.052	0.246	−0.007	0.002
Monthly income (Tk)	5.14 ± 0.001	0.056	0.179	<0.0001	<0.0001
HbA1_c_ %	−0.012 ± 0.008	−0.062	0.145	−0.028	0.004
Acquisition of information	−0.010 ± 0.036	−0.286	0.775	−0.082	0.061
Prescribed treatment	−0.022 ± 0.011	−0.084	0.048	−0.044	0.0001
Body mass index (kg/m^2^)	0.001 ± 0.002	0.022	0.586	−0.003	0.005

Adjusted R^2^ = 20 %; Overall model F test, p = 0.0001

*DM* diabetes mellitus

^a^Unstandardized sample regression co-efficient

^b^Standardized sample regression co-efficient

## Discussion

The quality of life is gradually gaining importance as the clinical or physiological outcome parameter. This study provides a perspective of HR-QoL among Bangladeshi patients with type 2 diabetes.

In the present study, more than half of the individuals with type 2 diabetes reported ‘some problems’ in the pain/discomfort (57.8 %) and anxiety/depression (59.4 %) dimensions whereas it was 44 % in the dimensions of mobility and usual activities and 27 % in self-care. The percentage in the dimensions of mobility, usual activities, and self-care was comparatively low compared to other two dimensions. This finding was inconsistent with the reports from Japan [8], Korea [9], and Singapore [10]. The percentages of Japanese patients reported problems were 21.2 % for mobility, 2.8 % for self-care, 17.3 % for usual activities, 35.7 % for pain/discomfort, and 19.7 % for anxiety/depression [8]. In Korea, some changes were observed in the HR-QoL among patients with diabetes [9]. Thirty percent of patients in Singapore had problems in pain/discomfort and anxiety/depression [10]. In Oman, patients with type 2 diabetes had the moderate HR-QoL [11]. The differences in the results with other populations may be due to the HR-QoL which is a time-dependent variable and should be repeatedly measured in patients with type 2 diabetes to ensure reliable estimations. Discontinuation of follow-up by patients, the quality of diabetes care, and the availability of access to support services could be the reasons of observing the change in the HR-QoL.

We also predicted that the HR-QoL decreases because of some other factors. Evidence shows that the impaired HR-QoL is associated with age [8, 9, 11–13] and gender [9, 12, 13]. In the present study, the female patients had a 3-5-time higher risk of less mobility and usual activities compared to the male patients.

In our study, income also played a significant role in the HR-QoL. The upper-middle- and lower-middle-income groups in particular had less mobility and usual activities, although they had problems in pain. However, income was not found as an important factor in other studies [8–13].

In our study, the duration of diabetes was significantly associated with the self-care domain only, and the results revealed that the increase in the duration of DM decreased self-care. Consistent with findings of a study, patients with type 2 diabetes of more than 5 years had less satisfaction in self-care [11].

Patients with a family history of DM were satisfied with the HR-QoL domains self-care and usual activity. A possible explanation could be that a previous family history of diabetes is likely to increase awareness of the disease among other members of the family. Patients get information from them about its management. However, social support and family history of DM empower the patient’s individual attitudes, with the end result of enhancing the quality of life and reducing the severity of illness.

In the present study, higher educated and rural patients had a good HR-QoL. Similarly, Omani patients with 6 years of education had a significantly better quality of life compared to other patients with lower level of education [11]. Education gives power to the patient for self-management and has become a cornerstone of quality-oriented diabetes care. In our society, we notice that rural people are hard-workers, and their lifestyle and dietary habits are still better (especially with respect to consuming more fresh vegetables and other foods; doing hard physical activities) compared to urban people. Simultaneously, our government has been providing healthcare facilities at the peripheral areas, and on the other hand, the electronic media have reached those areas. People of the peripheral areas who have access to media can also get information on diabetes and its related complications. These are the probable reasons for the satisfactory results in our study relating to the quality of life among the rural people.

Several studies have reported that obesity [9, 11, 13], type of treatment [10, 13], glycemic status [10, 11, 13], and complications/co-morbid conditions [9, 12, 13] are associated with the quality of life. In the present study, type of treatment, obesity, glycemic status, and acquisition of information were found to be associated with the HR-QoL but not significantly. This result indicates that, in addition to the parameters analyzed in the study, other factors, that need to be studied, were significantly affecting the quality of life in patients with type 2 diabetes and those might be the patients’ perspective, ideas, and expectations relating to management of diabetes. With poor glycemic control and higher BMI, the risk of developing diabetes-related complications increases, which causes physical well-being to deteriorate. Besides this, psychological and physiological effects, such as increased anxiety owing to frustration with the inability to manage the condition, are likely to affect one’s quality of life.

The study has some limitations. The study being a center and outpatient-based one, its results may not be truly representative of all DM patients. We had also no data on diabetes-related complications/co-morbid conditions. Since it was a cross-sectional study, it cannot, therefore, determine causality. Longitudinal studies assessing the natural history of diabetes and the quality of life are needed to draw a solid conclusion on the causal pathway of these associations. Equivalently, we could not collect data for a matching cohort, which could help us compare and find the impact of the disease on our study patients. In this study, the patients self-reported, and the results might not, thus, give the true reflection in all aspects.

The quality of life of patients is an essential factor that affects diabetic management. The present study provides basic information on the quality of life, and the findings indicate that patients with diabetes suffer from moderately poor HR-QoL. The study has several implications. The ultimate diabetic care should, therefore, include the assessment of quality of life in any modality used for treating diabetic patients.

Although this study generated baseline data relating to quality of life of diabetes patients in our country, more studies, with a larger sample-size and with matching cohort, are strongly needed in other parts of Bangladesh for identifying the weaknesses of diabetes management for observing the impact of the disease. To run successful healthcare programs for diabetes patients, we have to include physiotherapy and health educator facilities and also construct available counselors and counseling center. Further longitudinal studies will help researchers and policy-makers to create cartulary effective intervention programs and policy for diabetes patients in Bangladesh.

## Conclusions

The findings of the study suggest that most patients have problems in the domain of pain/discomfort and anxiety/depression, and half of the patients have problems in mobility and usual activities. Age, female gender, income, education, family history and duration of DM, prescribed treatment, and glycemic status are important factors associated with the HR-QoL in patients with type 2 diabetes.
